# ^1^H-NMR-Based Metabolic Profiling of Maternal and Umbilical Cord Blood Indicates Altered Materno-Foetal Nutrient Exchange in Preterm Infants

**DOI:** 10.1371/journal.pone.0029947

**Published:** 2012-01-23

**Authors:** Illa Tea, Gwénaëlle Le Gall, Alice Küster, Nadia Guignard, Marie-Cécile Alexandre–Gouabau, Dominique Darmaun, Richard J. Robins

**Affiliations:** 1 Elucidation of Biosynthesis by Isotopic Spectrometry Group, Unit for Interdisciplinary Chemistry, Synthesis-Analysis-Modelling (CEISAM), University of Nantes–CNRS UMR 6230, Nantes, France; 2 Institute of Food Research, Norwich Research Park, Colney, Norwich, United Kingdom; 3 UMR Physiologie des Adaptations Nutritionnelles (PhAN), INRA, CNRH, Nantes, France; 4 Faculty of Medicine, University of Nantes, Nantes, France; 5 Neonatology and INSERM, Centre d'Investigation Clinique (CIC), CHU de Nantes, Nantes, France; National Institute of Agronomic Research, France

## Abstract

**Background:**

Adequate foetal growth is primarily determined by nutrient availability, which is dependent on placental nutrient transport and foetal metabolism. We have used ^1^H nuclear magnetic resonance (NMR) spectroscopy to probe the metabolic adaptations associated with premature birth.

**Methodology:**

The metabolic profile in ^1^H NMR spectra of plasma taken immediately after birth from umbilical vein, umbilical artery and maternal blood were recorded for mothers delivering very-low-birth-weight (VLBW) or normo-ponderal full-term (FT) neonates.

**Principal Findings:**

Clear distinctions between maternal and cord plasma of all samples were observed by principal component analysis (PCA). Levels of amino acids, glucose, and albumin-lysyl in cord plasma exceeded those in maternal plasma, whereas lipoproteins (notably low-density lipoprotein (LDL) and very low-density lipoprotein (VLDL) and lipid levels were lower in cord plasma from both VLBW and FT neonates. The metabolic signature of mothers delivering VLBW infants included decreased levels of acetate and increased levels of lipids, pyruvate, glutamine, valine and threonine. Decreased levels of lipoproteins glucose, pyruvate and albumin-lysyl and increased levels of glutamine were characteristic of cord blood (both arterial and venous) from VLBW infants, along with a decrease in levels of several amino acids in arterial cord blood.

**Conclusion:**

These results show that, because of its characteristics and simple non-invasive mode of collection, cord plasma is particularly suited for metabolomic analysis even in VLBW infants and provides new insights into the materno-foetal nutrient exchange in preterm infants.

## Introduction

The human foetus triples its weight during the last trimester of pregnancy, and is entirely dependent on ‘imported’ nutrients to cover its protein and energy needs. As protein accretion is a prerequisite for growth, the supply of amino acids from the maternal circulation is crucial to support foetal growth. The transfer of amino acids across the placenta from maternal to foetal circulations is complex [1] as three barriers are involved: nutrients are first taken up from the maternal circulation across the microvillous membrane, diffuse through the cellular cytoplasm, and finally are transported across the placental basal membrane into the umbilical circulation [2]. The first step of the transfer of amino acids and glucose through the placenta has been well studied but the transfer towards the foetus is less well described [2]–[4]. Understanding this process and its regulation will help develop interventional nutritional strategies, particularly in situations such as intrauterine growth restriction (IUGR) or preterm delivery when materno-foetal transfer is impaired (see [1] and refs therein) or foetal substrate synthesis is inadequate [5], [6].

During normal pregnancy, the concentration of most amino acids is higher in foetal than in maternal plasma. Several studies [2], [6]–[9] have shown that overall amino acid concentration is decreased in foetuses with IUGR, particularly for branched-chain amino acids, in spite of a concomitant elevation in maternal plasma concentrations. Accordingly, the rate of materno-foetal amino acid transfer, which can be assessed with techniques exploiting the infusion of stable-isotope-labelled amino acids, is reduced in IUGR infants, compared with control babies [10] and the drop in the materno-foetal isotope enrichment ratio depends on the severity of IUGR [11]. Amino acid transfer was also found to be affected by prematurity [12], maternal diabetes [13] or hypoxia [14].

In addition, deficiencies in maternal plasma can obviously affect foetal nutrient supply, and it is essential to establish whether insufficiency is related to altered materno-foetal supply or to foetal metabolism. As an example, we have recently shown that very low birth weight (VLBW) preterm neonates possess a fully active capacity to synthesize glutathione (GSH), a key antioxidant, and that the observed depletion in umbilical blood may arise from inadequate cysteine availability from the maternal venous blood supply to the placenta [15].

Most studies of materno-foetal nutrient gradients have focused on specific nutrients, and do not provide a full-picture of nutrient supply to the foetus. Apart from amino acids [1], [10]–[14], the materno-foetal exchange of other essential nutrients has been poorly explored. Furthermore, transfer across the placenta of other substrates, such as those involved in energy metabolism, has not been adequately investigated.

To get further insight into the materno-foetal nutrient transfer and assess metabolic profiles of the VLBW neonates and their mothers, a metabolomic approach based on nuclear magnetic resonance (NMR) spectroscopy has been adopted, as we believe this offers the best prospect to highlight potential metabolic deregulation in VLBW infants. The advantage of using high resolution ^1^H NMR metabolomic profiling is that it is possible to assess the main plasma proton-containing metabolites even at sub-mM concentrations in a body fluid, with an excellent precision and reproducibility [16].

The most commonly used biological fluids sampled for metabolomics studies are urine, blood plasma or serum. However, the use of non-invasive methods is an essential requirement in neonatal medicine, especially in studying VLBW infants. Although the metabolomic analysis of urine has been used to assess the overall metabolic status of term and preterm neonates [17], [18] and is appropriate to explore postnatal metabolic maturation, it does not provide any insight into prenatal metabolite supply and materno-foetal substrate transfer at birth. One previous study using ^1^H NMR spectroscopy to examine plasma collected from umbilical and maternal veins of mothers delivering healthy normo-ponderal full-term (FT) neonates has been reported [19]. While this study surveyed the presence of lactate, alanine, valine, and lipoproteins in maternal and cord venous blood, neither umbilical arterial blood plasma nor the effect of VLBL were analyzed.

The working hypothesis adopted is that there will be general changes that can be associated with preterm natural delivery. In order to seek general metabolic differences between term and preterm deliveries, we have included in our study VLBW group subjects with a known state of disease (diabetes), with pregnancy-induced hypertension and with no evident identified cause of premature delivery. We also include natural delivery and delivery by caesarean section. Hence, we aim to obtain insight into the materno-foetal metabolite exchange without any particular bias. For this group, and a control group of FT normal delivery subjects, we report a metabolomic analysis on plasma from three sources: the umbilical vein, which provides information about blood directed from the placenta to the foetus; one of the two umbilical arteries, representing blood of the foetus redistributed to the placenta; the maternal venous blood collected at delivery, in order to relate information obtained from umbilical blood to the status of the mothers.

## Materials and Methods

### Patients

The study was carried out on 8 VLBW infant-mother pairs and 8 control FT infant-mother pairs. The size of the population, while small, proved adequate to identify a number of biomarkers (see below). In view of the difficulty of recruiting members of the VLBW test set, due to both the unpredictability of premature birth and to the ethical requirement of not perturbing the mother at this stressful moment, we did not extend this initial study further.

The study group consisted of:

- a test set of 8 inborn very-low-birth-weight (VLBW) neonates. Inclusion criteria were a gestational age (GA) <32 weeks and/or a birth weight <1500 g. The gestational age was between 6 and 14 weeks premature. Exclusion criteria were: perinatal asphyxia or major foetal pathology (abnormal karyotype, malformation, foetal pathology revealed during pregnancy);
- a control set of 8 inborn normo-ponderal full term (FT) neonates. Inclusion criteria for the control group were: a GA >37 weeks, and an uneventful pregnancy and delivery. Exclusion criteria for the control group were: perinatal asphyxia, major foetal pathology, bacterial or viral infection, or maternal arterial blood pressure >90 mm Hg during pregnancy;
- the 16 mothers of the VLBW and FT infants.

**Table 1** gives the clinical parameters for the 16 neonates and their mothers.

**Table 1:** Selected clinical characteristics of enrolled infants and their mothers.

	Group		
	Preterm VLBW subjects	FT subjects	
	(n = 8)	(n = 8)	p†
Sex			
Male, *n*	3 (37%)	3 (37%)	
Female, *n*	5 (63%)	5 (63%)	
Birth weight, *g*	1180 [940–1351]	3320 [3098–3429]	<0.001
Gestational age, *wk*	28.9 [27.4–30.0]	40.2 [39.6–41.0]	<0.001
Birth weight, *z score*			
pH in umbilical cord blood	7.3 [7.2–7.4]	7.3 [7.2–7.4]	
Mode of delivery			
Vaginal, *n*	3 (37%)	8 (100%)	
Caesarean section, *n*	5 (63%)	0 (0%)	
Preeclampsia, *n*	3 (37%)	0 (0%)	
Apgar score at 5 min	10 [8]–[10]	10 [10–10]	0.06
Infant haemoglobin *g/dl*	15.5 [14.3–17.5]	17.5 [14.7–18.1]	0.88
Maternal age, *y*	31.7 [27.7–36.1]	28.8 [27.1–32.1]	0.34
Maternal haemoglobin *g/dl*	11.6 [10.7–12.1]	12.7 [12.2–13.0]	0.02
Maternal hypertension	5 (63%)	0 (0%)	

Data are reported as n (%) or median [interquartile 25–75].

† Inter-group comparison by Mann-Whitney U test.

Written, informed consent was obtained from each mother a few hours before delivery, according to a protocol approved by the local medical ethical committee (Comité de Protection des Personnes dans la recherche biomédicale (CPP) des Pays de la Loire).

The study was registered (ClinicalTrials.gov Identifier: NCT00607061), and performed in the Department of Obstetrics of the Hospital Mere-et-Enfant at the University of Nantes, Nantes. We confirm that our ethics committee specifically approved this study.

### Sample collection and preparation

Samples of arterial and venous umbilical cord blood were obtained immediately after birth. The sampling only used umbilical cord blood and did not involve any blood loss for the neonates. Maternal venous blood was collected at delivery for each mother-neonate pair included in order to obtain materno-infant pairs for comparison between the preterm delivery (VLBW) and the control (FT) groups. Samples were collected in chilled 5-mL EDTA-coated tubes. The plasma was separated immediately by centrifugation (5000 *g*, 10 min, 4°C) and stored at −20°C until required for NMR analysis.

### ^1^H NMR spectroscopy measurement of maternal and umbilical arterial and venous plasma

Samples were prepared from a plasma volume of 150 µL and 450 µL of a mixture of H_2_O/D_2_O (70/30% volume) in a 5 mm NMR tube.

High-resolution NMR spectra of all blood plasma were recorded at 303 K on a Bruker Avance DRX-500 spectrometer (Bruker, Karlsruhe, Germany), operating at 500.13 MHz for proton and equipped with a cryogenic probe. Standard methods for the acquisition of plasma NMR spectra were used [20]. 1D ^1^H NMR spectra were acquired using the NOESYPR1D (1D Nuclear Overhauser effect spectroscopy with water pre-saturation) pulse sequence (RD-90°-t_1_-90°-t_m_-90°-acquire) with a relaxation delay (RD) of 2 s, a mixing time (t_m_) of 150 ms and a fixed t_1_ delay of 20 µs. Water suppression was achieved by pre-saturation during the relaxation delay and mixing time. Each spectrum consisted of 128 free induction decays (FIDs) collected into 32K complex data points with a spectral width of 8012.8 Hz and an acquisition time of 2 s.

1D relaxation-edited ^1^H NMR spectra were acquired using the water suppressed Carr-Purcell-Meiboom-Gill (CPMG) spin echo pulse sequence (RD-90°-{τ-180°- τ}*_n_*-acquire) with a relaxation delay of 2 s and a total spin-spin relaxation time (2 *_n_* τ) of 200 ms in order to attenuate broad signals from proteins and lipoproteins. 256 FIDs were collected into 32K complex data points.

Prior to Fourier transformation, the FIDs were zero-filled to 64K points and multiplied by an exponential line-broadening function of 0.3 Hz. The 1D spectra were manually phased, baseline corrected, and the chemical shifts referenced internally to the α-glucose signal at δ 5.234 ppm, using Topspin™ software (Bruker, Karlsruhe, Germany).

### Data processing and chemometric analyses

Each ^1^H NMR spectrum of NOESYPR1D and CPMG sequences over the range (δ 0.7–8.5 ppm) was reduced to 70 and 92 segments, respectively. Using AMIX™ software (version 3.8.4, Bruker, Karlsruhe, Germany), segments were defined manually so as each to contain a single metabolite. Segments thus defined covered 99.5% of the spectrum. Signal intensity in each segment was integrated using AMIX software. Data was normalized in AMIX by dividing each integrated segment by the total area of the spectrum to reduce any significant concentration difference. Output data in ASCII data format was imported to Excel 2003 (Microsoft Corporation, Redmond WA, USA), mean centred and then exported to SIMCA-P+ (version 12.0, Umetrics, Umeå, Sweden) for statistical analyses.

### Statistical analysis of ^1^H NMR spectroscopic data

For each set of spectra (NOESYPR1D and CPMG), a data matrix was built, where each row corresponds to a sample, each column to a chemical shift and each value in the data table to the sum of the intensities of an NMR segment. The water region (δ 4.5–5.0 ppm) and the segments containing EDTA-related signals (δ 2.53–2.58, 3.1–3.3, 3.6–3.7 ppm) were excluded. Chemometric analysis was performed using SIMCA-P+. Initial data analyses were conducted using the unsupervised method of Principal Component Analysis (PCA). The NMR variables responsible for the differences between sample scores in the score plot can be detected in the corresponding loading plot [21]. In addition, a supervised method, Partial Least Squares Discriminant Analysis (PLS-DA), was applied to maximize the discrimination between sample groups focusing on differences according to preterm subject metabolic variations [22].

The quality of the PLS-DA models obtained was evaluated by three parameters: R^2^(X), corresponding to the proportion of the total variance of the dependant variables that is explained by the model, R^2^(Y), defining the proportion of the total variance of the response variable (i.e. the class of the samples) explained by the model, and the predictive ability parameter Q^2^(Y), which is similar to R^2^(Y) except that it is computed by cross-validation. In addition, a permutation test (*n* = 20) was carried out to validate and to test the degree of over fitting for PLS-DA model. The correlation coefficient between the original Y and the permuted Y is plotted against the cumulative R^2^ and Q^2^ and a regression line is calculated with the R^2^- and Q^2^-intercept limits. The model was successfully validated when R^2^(Y) and Q^2^(Y)>0.6 and observed intercept values of R^2^(Y) and Q^2^(Y)<R^2^(Y) and Q^2^(Y), respectively. The score values from PLS-DA were subjected to ANOVA to test the PLS-DA model and the validation was considered successful with p<0.05.

The variables which discriminate most significantly the metabolic signatures were pinpointed by their loadings on PLS-DA. The significance of the differences was further assessed by comparing the area normalized intensity of metabolite signals between the preterm and full-term plasma samples with the non parametric Mann Whitney U test. The critical *p* value was set at 0.05.

## Results

### Feasibility of ^1^H NMR spectroscopy for the analysis of arterial and venous umbilical cord plasma and maternal plasma

**Figure 1:**
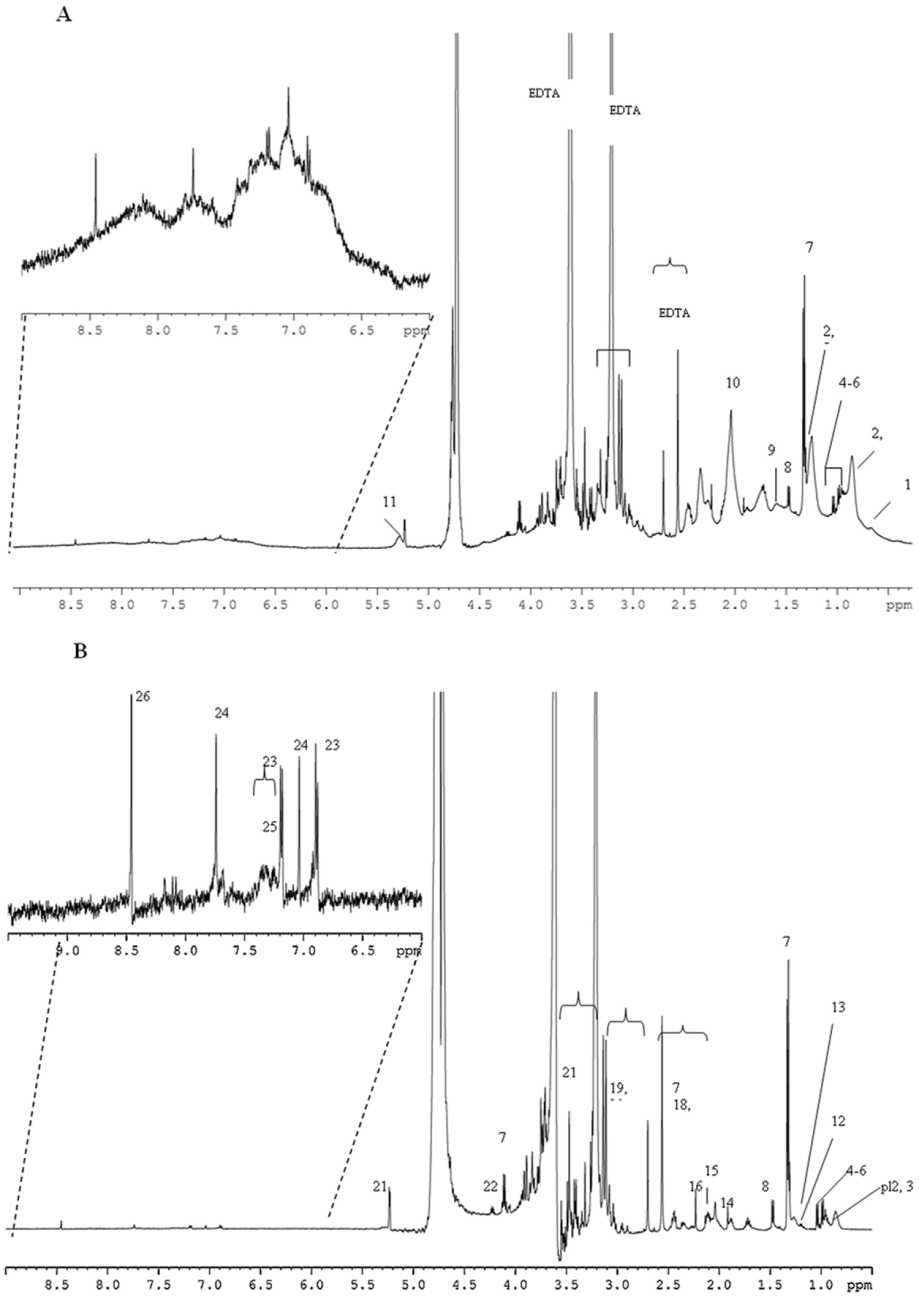
500 MHz ^1^H NMR spectra of arterial umbilical cord plasma from a VLBW delivery. (A) standard NOESYPR1D spectrum; (B) 1D relaxation–edited (CPMG) spectrum. Assignment: 1, HDL; 2, LDL; 3, VLDL; 4, valine; 5, leucine; 6, isoleucine; 7, lactate; 8, alanine; 9; lipid; 10, *N*-acetyl glycoprotein; 11, unsaturated lipid; 12, *β*-hydroxybutyrate; 13, lipid; 14, acetate; 15, glutamine; 16, acetone; 17, pyruvate; 18, succinate; 19, albumin-lysyl; 20, creatinine; 21, glucose; 22, threonine; 23, tyrosine; 24, methylhistidine; 25, phenylalanine; 26, formate.

Ethical constraints limit both the number of samples and the volume of blood that can be used for studies in new-born infants. However, samples of umbilical venous and umbilical arterial blood taken at parturition represent the placental supply to the foetus, and foetal arterial blood, respectively. Although it is difficult to obtain more than 150 µL of placental arterial blood plasma, this volume proved sufficient to obtain ^1^H NMR NOESYPR1D and CPMG spectra of arterial umbilical cord plasma in VLBW infants, as illustrated in **Figure 1A and 1B**. Similarly, spectra were also obtained from 150 µL samples of arterial umbilical cord plasma collected from FT infants at birth and from maternal venous plasma and venous umbilical cord plasma, both for FT and VLBW deliveries (spectra not shown). Such small volumes of untreated plasma samples (150 µL) proved sufficient for the detection and estimation of a wide range of metabolites in FT and VLBW infant plasmas.

### Metabolite profiles of arterial and venous umbilical cord plasma and maternal plasma obtained by ^1^H NMR spectroscopy

Based on the literature [23], [24], twenty six metabolites were identified from the two spectral types acquired, NOESYPR1D (**Figure 1A**) and CPMG (**Figure 1B**). As typically observed, the NOESYPR1D spectrum is dominated by the broad resonances of lipoproteins and other plasma proteins, which mask the signals from low-molecular-weight metabolites. In contrast, by employing the CPMG pulse sequence to attenuate or even eliminate resonances from macromolecules (or bound small molecules) with shorter relaxation times [23], [25], the spectrum acquired (**Figure 1B**), gives a clear representation of the low-molecular-weight metabolites present, especially in the low-frequency (δ 0–3 ppm) and high-frequency (δ 6–10 ppm) regions of the spectrum. The metabolites identified in **Figure 1** cover a typical range of low molecular weight compounds found in plasma, including amino acids, glycolysis metabolites, ketone bodies, and higher molecular mass metabolites including lipoproteins, unsaturated lipids, glycoproteins, and albumin-lysyl, as indicated by the signals corresponding to the *N*-acetyl groups and lysyl groups, respectively.

Venous umbilical cord plasma (450 µL) and maternal plasma taken at FT delivery have previously been examined by ^1^H NMR spectrometry and some of these metabolites identified [19]. In this earlier study, the Hahn spin echo sequence was used, which did not allow signals in the aromatic region to be detected. Nevertheless, metabolites such as *β*-hydroxybutyrate, valine, alanine, glutamine and the *N*-acetyl groups of glycoproteins from plasma proteins were identified [19]. Arterial umbilical cord blood plasma, which represents blood supply from the foetus, was not examined by these authors. In fact, it is relatively difficult to obtain a sufficiently large sample of arterial cord blood for ^1^H NMR spectroscopy. In the present study, we worked with a sample size of only 150 µL and showed that it is possible therein to identify simultaneously 26 metabolites (**Figure 1A and 1B**). Thus, three plasmas obtained from three different sites –arterial and venous umbilical cord blood and maternal venous blood –can be studied under comparable conditions, indicating that ^1^H NMR spectrometry may be an exploitable tool in neonatal research.

## Discussion

### Clear-cut differences are observed between umbilical arterial, umbilical venous and maternal plasma by PCA

**Figure 2:**
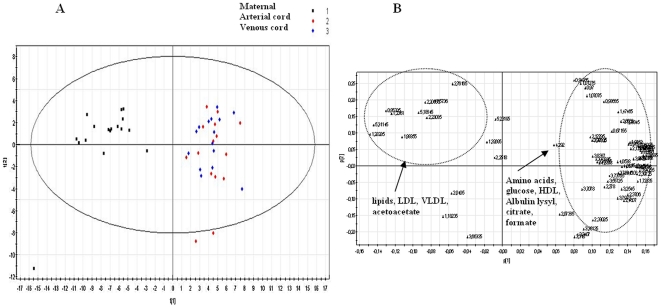
Multivariate analyses resulting from the 48 standard 1D spectra of maternal and both venous and arterial cord plasma from FT and VLBL deliveries. (A): PCA scores plot. (B): PCA loadings plot. The maternal plasma and both venous and arterial cord plasma are clearly distinguished on the first component which accounts for 54% of the total variance. The second component, which account for 14% of the total variance is related to inter-individual differences.

Plasma samples collected at the three different sites for VLBW preterm and FT deliveries were assessed using PCA analysis to determine the most important sources of variability between the samples collected from the three different blood origins. The scores scatter plot resulting from applying PCA to the NOESYPR1D spectra (water and EDTA signals excluded) is shown in **Figure 2A**. Regardless of gestational age, a clear separation between maternal and venous/arterial umbilical cord plasma was observed along principal component 1 (PC1 54%). **Figure 2B** represents the loading plot corresponding to the first two PCs of the PCA model. The metabolic variations based on the PCs loading orientations showed higher levels of amino acids, glucose, HDL, albumin-lysyl and lower levels of lipids, LDL, VLVL, *N*-acetyl groups of glycoproteins, acetoacetate, and *β*-hydroxybutyrate in both arterial and venous umbilical cord plasma compared with maternal plasma.

**Figure 3:**
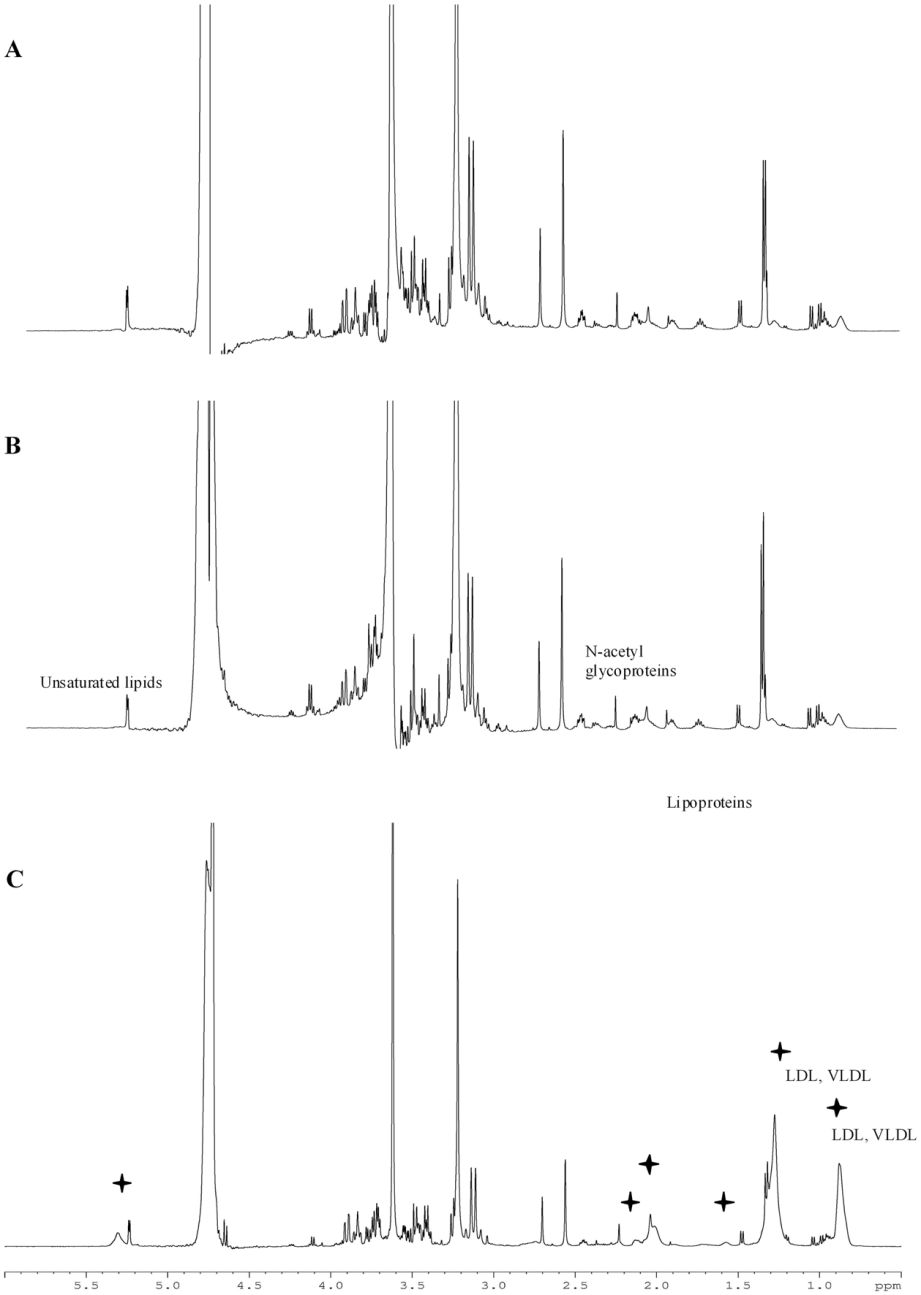
500 MHz 1H NMR CPMG spectra of blood plasma samples from VLBW deliveries. (A) Arterial cord blood, (B) Venous cord blood (C) Maternal blood. The same spectral profile was obtained for arterial and venous blood plasma, whereas the spectrum of maternal blood plasma was different. The differences are marked by +.

**Figure 4:**
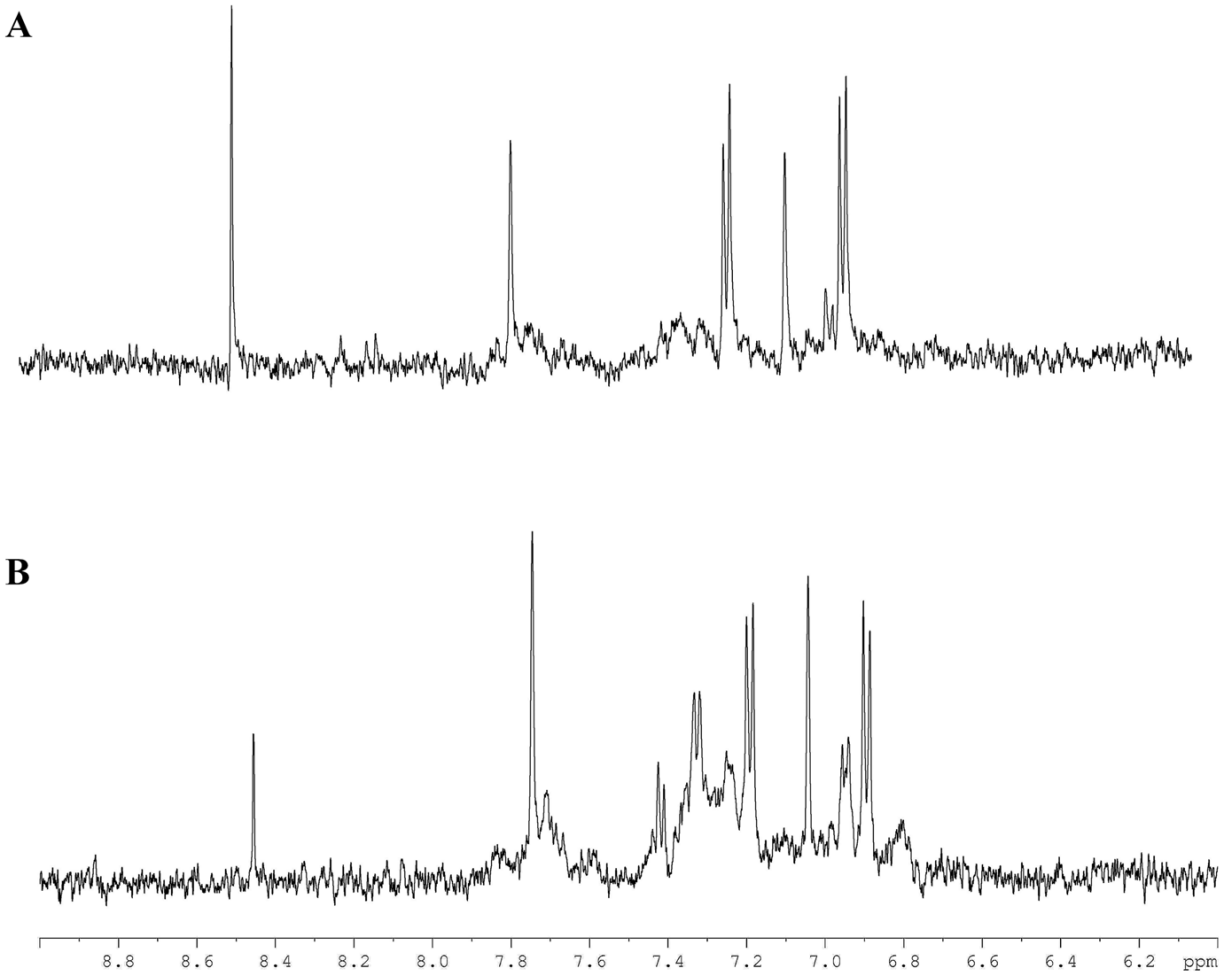
500 MHz 1H NMR CPMG spectra for the aromatic region of blood plasma samples from a VLBW delivery. (A) Arterial cord blood, (B) Maternal blood. The maternal plasma shows signals at 6.80, 6.93, 7.23 and 7.68 ppm that are absent from the preterm plasma.

**Figure 3** illustrates ^1^H NMR metabolomic spectra of umbilical arterial (**Figure 3A**), umbilical venous (**Figure 3B**) and maternal (**Figure 3C**) plasma from a mother-neonate pair delivery at preterm (VLDW). As inferred from **Figure 2A**, there was no noticeable difference between spectra from arterial and venous umbilical cord plasma. In contrast, the spectrum of the maternal plasma is dominated by a broad envelope of protein and lipid resonances, virtually absent in the umbilical plasma samples. Signals are assigned to LDL, VLDL, lipoproteins, *N*-acetyl groups of glycoproteins and unsaturated lipids. Additionally, the aromatic region of the maternal plasma spectrum (δ 6–9 ppm) also shows marked differences from both the arterial and venous umbilical cord plasmas (**Figure 4A**), with an increase of signals at 6.80, 6.93, 7.23, 7.68 ppm (**Figure 4B**). These signals are probably due to the presence of macromolecules, such as lipids and/or proteins.

These results confirmed those found in previous studies by classical techniques, where an elevation of plasma lipids was reported in the plasma of women during uncomplicated pregnancy, with an increase in triglyceride, total cholesterol, VLDL, HDL and LDL levels [26]–[28], along with lipid levels in foetal umbilical cord and neonatal plasma below those reported in adults [29], [30], meeting maternal [31] and developing foetal [32] energy requirements in late gestation.

Amino acid concentrations have been known to be significantly higher in foetal than in maternal blood, considered to reflect the presence of active transport systems within the placenta [1]. The placenta also has the capacity for utilization, production, and interconversion of amino acids, all of which can profoundly affect the quantity of an amino acid delivered into the foetal circulation.

**Figure 5:**
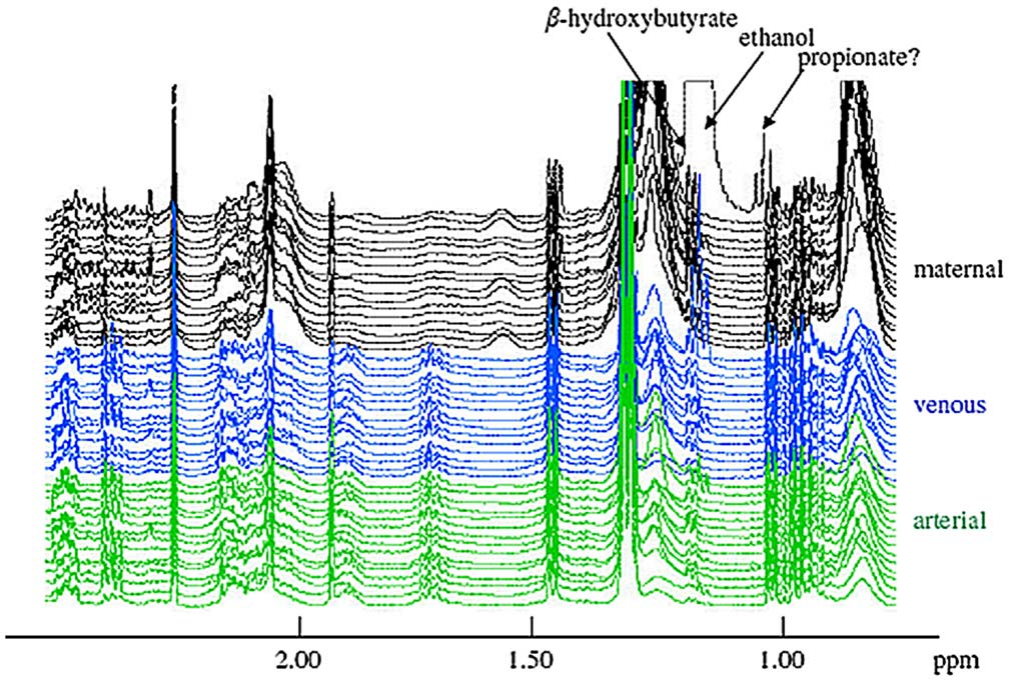
500 MHz ^1^H NMR CPMG spectra of maternal, cord venous and cord arterial plasma. Note the presence of traces ethanol in most spectra and high ethanol and possibly propionate (1.05 ppm) in one unusual maternal profile (the top one). This figure also shows the differences in lipids between maternal and cord plasma (higher levels of lipid signals at 0.90, 1.23, 1.72 and 2.00 ppm) and in lysine levels (signals at 1.71 and 1.90 ppm higher in maternal plasma).

Among the 16 analysed maternal plasmas, one sample displayed an unexpected biochemical profile, showing the most negative scores (outlier at the bottom left of the score plot **Figure 2A**). When this spectrum was scrutinised, triplet and quadruplet resonances at 1.18 and 3.65 ppm, for the methyl and methylene groups of ethanol were unexpectedly detected (**Figure 5**) accompanied by another triplet at 1.05 ppm which was unique to this ^1^H NMR spectrum (possibly the methyl group of propionate). Contamination during handling was ruled out as a potential source of ethanol. This atypical biochemical profile also showed a disturbed lipid composition and a depletion of amino acids in the maternal plasma. Intriguingly, however, neither an enhanced level of ethanol nor any other evident change was seen in the arterial and venous umbilical cord plasma from this mother–infant pair. The same preservation in cord plasma amino acid concentrations was observed in the plasma from rat pups born from dams that had a severe diet-induced depletion of total plasmatic amino acids [33]. This finding is consistent with the previous study of venous umbilical cord plasma by ^1^H NMR, wherein ethanol was identified in 5 venous umbilical cord plasma samples, suggesting ethanol to be endogenous. In fact, a majority of the ^1^H NMR spectra from both maternal and cord blood showed traces of ethanol (**Figure 5**). Endogenous ethanol is known to be produced in small quantity in mammalian tissue [34] via the reduction of acetaldehyde, itself derived from the decarboxylation of pyruvate [35]. This finding is consistent with the hypothesis that endogenous ethanol is produced as an intermediate step for the elimination of excess energy-releasing substrates from mitochondria [36], [37] as by the human body during intense physical effort.

### Effect of preterm delivery on maternal plasma metabolomics

**Figure 6:**
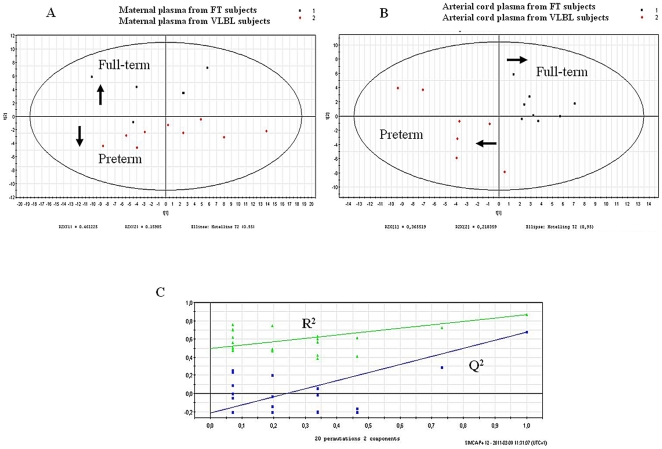
Multivariate analyses resulting from the standard 1D 500 MHz ^1^H NMR spectra from FT and VLBW preterm deliveries. (A): PCA scores plot resulting from the analysis of maternal plasma. (B): PLS-DA scores plot resulting from the analysis of arterial umbilical cord plasma and validation by permutation test (C). (C): R^2^(X) = 0.366, R^2^(Y) = 0.695 and Q^2^(Cum) = 0.676. The model has been validated by a permutation test (n = 20). R^2^ intercept is at 0.495 and Q^2^ intercept is at −0.212.

Analysis by PCA of the ^1^H CPMG NMR spectra of plasma collected from mothers delivering either FT or VLBW infants reveals differences between the two groups (**Figure 6A**). The metabolites responsible for these differences were assessed with the loading plot from PCA (data not shown) and by univariate statistical tests of each metabolite (**Table 2**). Compared with mothers delivering at FT, mothers delivering VLBW infants had significantly higher levels of lipids, pyruvate, glutamine, valine and threonine, and significantly lower levels of acetate and isoleucine. Although not statistically significant (p = 0.08), there was an increase in the *N*-acetyl signals from the glycoproteins in the blood of preterm mothers (**Table 2**). These signals arising from acute phase glycoproteins [38] are known to reflect inflammatory status [39], [40].

**Table 2:** Maternal and cord blood plasma metabolite differences between full-term and VLBW subjects.

	Maternal plasma			Venous cord plasma			Arterial cord plasma			Gradient M-V		
Metabolite chemical shift ppm	FT	VLBW	p†	FT	VLBW	p†	FT	VLBW	p†	FT	VLBW	p†
**Lipoproteins**												
HDL 0.67§	0.60	0.61	0.70	0.74	0.68	0.02*	0.74	0.66	0.05*	−0.11	−0.06	0.11
LDL 0.83	2.27	2.72	0.30	1.16	0.87	0.02*	0.98	0.67	0.04*	1.11	1.82	0.16
lipids 1.25	4.65	7.72	0.01#	1.88	1.43	0.30	1.56	1.11	0.20	2.77	5.81	0.03*
lipids 1.3	5.37	8.06	0.03*	1.96	1.46	0.35	1.56	1.23	0.42	3.41	5.97	0.06
VLDL 1.57§	1.80	1.83	0.85	1.67	1.59	0.02*	1.67	1.59	0.01#	0.12	0.22	0.56
unsaturatedlipids 5.31	0.41	0.64	0.03*	0.20	0.10	0.05*	0.16	0.17	0.91	0.21	0.49	0.02*
**Glycolysis pathway**												
Lactate 1.32§	6.96	6.96	0.50	6.28	7.14	0.42	6.52	7.47	0.35	0.67	−0.33	0.20
Acetate 1.91	0.62	0.31	0.01#	0.43	0.51	0.16	0.70	0.58	0.56	0.19	−0.20	0.03*
Pyruvate 2.37	0.31	0.61	0.00#	0.80	0.64	0.05*	0.80	0.55	0.04*	−0.48	−0.03	0.00#
Glucose 3.7	4.46	3.67	0.21	5.81	3.52	0.01#	5.31	2.57	0.01#	−1.79	0.26	0.01#
Glucose and/or glycerol 3.87	0.89	0.90	0.77	1.01	0.98	0.56	1.01	0.90	0.02*	−0.13	−0.07	0.16
Formate 8.45	0.02	0.02	0.44	0.03	0.06	0.02*	0.07	0.08	0.64	−0.01	−0.04	0.08
**Ketone bodies**												
3-Hydroxybutyrate 1.20	2.87	1.06	0.12	0.91	0.57	0.11	0.60	0.42	0.06	1.96	0.41	0.35
Acetone 2.23	1.47	1.28	0.29	1.62	1.02	0.00#	1.39	0.86	0.01#	−0.15	0.20	0.30
**Proteins**												
N-Acetyl-glycoprotein 2.04	2.64	3.14	0.08	1.76	1.49	0.06	1.61	1.22	0.04*	0.88	1.57	0.08
Albumin-lysyl 2.9§	0.48	0.44	0.21	0.88	0.81	0.01#	0.90	0.84	0.13	−0.29	−0.25	0.56
Albumin-lysyl 3.01§	0.78	0.73	0.29	1.31	1.21	0.06	1.32	1.20	0.03*	−0.57	−0.48	0.49
creatinine 4.05§	0.53	0.59	0.03	0.77	0.74	0.42	0.79	0.68	0.03	−0.24	−0.16	0.08
**Amino acids**												
**Non-essential**												
Alanine 1.47	0.67	0.72	0.85	1.26	1.11	0.20	1.20	0.81	0.04*	−0.59	−0.38	0.11
Glutamine/glutamate 2.13§	1.10	1.33	0.01#	1.46	1.62	0.05*	1.42	1.42	0.56	−0.28	−0.25	0.73
Glutamine 2.45	0.36	0.51	0.05*	0.43	0.77	0.00#	0.43	0.67	0.03*	−0.07	−0.26	0.04*
Tyrosine 6.89§	0.21	0.20	0.15	0.36	0.35	0.35	0.37	0.33	0.01#	−0.15	−0.14	0.91
Tyrosine 7.18§	0.24	0.24	0.85	0.41	0.39	0.35	0.42	0.37	0.01#	−0.17	−0.15	0.49
**Essential**												
Valine 0.98	0.59	0.86	0.00#	1.11	1.27	0.25	1.03	0.95	0.35	−0.53	−0.45	0.91
Valine 1.03§	1.39	1.38	0.21	1.73	1.73	0.91	1.73	1.63	0.04*	−0.34	−0.11	1.00
Isoleucine 1.01§	1.54	1.50	0.03*	1.69	1.61	0.16	1.69	1.52	0.03*	−0.14	−0.11	0.82
Leucine 1.71§	0.18	0.20	0.44	0.37	0.39	0.49	0.39	0.31	0.11	−0.19	−0.20	0.64
Threonine 4.24§	0.39	0.44	0.04*	0.56	0.51	0.08	0.58	0.46	0.01*	−0.17	−0.08	0.06
Methylhistidine 7.74§	0.14	0.15	0.50	0.27	0.25	0.06	0.28	0.23	0.00#	−0.12	−0.09	0.04*

Values are given as area normalized peak signals mean (n = 8 for each group).

§ designates spectral signals integrated from NOESY spectra, all other signals being integrated from CPMG spectra.

† Significant differences at p<0.01 and p<0.05 are designated by # and *, respectively.

### Effect of gestational age on venous umbilical cord plasma of FT and VLBW infants

No significant overall differences between venous umbilical cord plasma collected from mothers delivering at term or prematurely were revealed by PLS-DA of the ^1^H CPMG or NOESYPR1D NMR spectra. However, univariate statistical testing indicated some biomarkers to differ significantly. As shown in **Table 2**, significant decreases in levels of lipids, HDL, VLDL, LDL, pyruvate, glucose, acetone, albumin-lysyl, and significant increases in levels of formate and glutamine/glutamate were observed in venous umbilical cord plasma collected from VLBW preterm delivery. A lower level of lysyl groups of albumin is indicative of a degree of oxidative stress [41]. It has recently been suggested that normal pregnancy is associated with a level of relative hypoxia with the addition of reperfusion and oxidative stress [42], and the amounts of albumin-lysyl may reflect increased oxidative stress in the case of the VLBW materno-foetal unit, despite the prevalence of caesarean delivery. Note that more than half the VLBW group suffered from hypertension (**Table 1**). Although the levels of glucose were similar in the plasma of mothers delivering at FT and preterm (with a trend on lower levels for the latter), glucose levels were 1.5-fold lower in the venous cord plasma of VLBW compared with FT neonates, and 2-fold lower in arterial cord plasma (see next section). Interactions between maternal glucose and foetal growth are not completely understood but there may be a causal link between low maternal blood glucose and low birth weight [43].

### Biochemical alterations in arterial umbilical cord plasma of FT and VLBW infants

Analysis of ^1^H NOESYPR1D spectra from all arterial plasma samples by PLS-DA revealed metabolic variations related to the term of delivery (**Figure 6B**). Most metabolite levels in arterial umbilical cord plasma from preterm infants were significantly different from those in venous umbilical cord plasma (**Table 2**). Significant decreases in the level of HDL, LDL, VLDL, pyruvate, glucose, acetone, albumin-lysyl, alanine, tyrosine, valine, isoleucine, leucine, threonine and 3-methyl-histidine were observed in arterial cord plasma from VLBW infants, accompanied by an increase in glutamine levels. Hence, it is apparent that the ^1^H NMR spectra obtained made it possible to identify several biomarkers associated with VLBW infants. As for the venous cord plasma, the levels of the albumin-lysyl residues were noticeably lower in arterial umbilical cord plasma samples from preterm delivery, indicating again the presence of foetal oxidative stress. This result is in agreement with the known depletion in GSH documented in VLBW infants compared with full-term neonates [44]. Glutamine levels were elevated in both the venous and arterial cord blood plasma of VLBW neonates: the reason for this is not clear. It is noteworthy, however, that glutamine is the most abundant amino acid in blood and is involved in multiple pathways including the biosynthesis of purines and pyrimidines [45], and the supply of energy to neonatal gut [46] and to other rapidly dividing cells. Moreover, glucocorticoids, which are secreted in excess during stressful situations, have been shown to enhance both the *de novo* synthesis and the utilization of glutamine [47]. As preterm birth is associated with significant stress, we speculate that the increase of glutamine reflects a dramatic surge in glutamine biosynthesis and placental transfer in order to cover the need of the stressed preterm infant. Increased rates of glutamine production have been reported in VLBW infants [46], [48], and higher levels of glutamine were observed in the amniotic fluid of malformed foetuses [49]. Most essential and non-essential amino acids were depleted in VLBW plasma compared with FT plasma (**Table 2**). Placental amino acid transport can be reduced due to either impaired foetal and placental growth or to decreased transporter concentrations [1]. Decreased transporter capacity has been found in human IUGR placental vesicles [50], bearing out *in vivo* studies that indicate decreased transport in IUGR pregnancies [10], [11].

### Maternal-to-venous gradients for FT and VLBW infants

Univariate statistical tests were performed on the values of nutrient gradients for maternal-to-venous (M-V) umbilical cord plasma of FT and VLBW infants. Negative values of these gradients reflect a net transfer from maternal circulation to the foetus, whereas positive values mean that the transfer from the mother to the foetus may be deficient. M-V gradients differed significantly between VLBW and FT infants (**Table 2**). Significant increases of HDL, lipids, glucose and histidine were observed in preterm infants, showing a weak transfer of these metabolites from the mother to the foetus. A low transfer of lipids may reflect the limited adipose storage of the foetus until the third semester of the pregnancy [51], rather than an increase of *β*-oxidation in foetal tissues due to a greater use of free fatty acids, as the foetus is believed to cover its energy needs solely with the oxidation of glucose.

In agreement with previous studies showing the existence of active glutamine transport from the maternal compartment to foetal blood [1], [2], a decrease in the glutamine gradient was observed in both groups, indicative of the utilization of this metabolite by the placenta. Placenta is known to transaminate other amino acids such as leucine, isoleucine and valine to their respective *α*-ketoacids, which are released into the umbilical and uterine circulation and contribute to placental ammonia production, consistent with the high level of *β*-hydroxybutyrate and acetate found in maternal plasma.

### Conclusions

Based on a detailed analysis of ^1^H NMR spectra from maternal plasma and both venous and arterial umbilical cord blood plasma, a number of metabolites can be shown to vary depending on the term of delivery. In particular, profiles of both maternal and arterial umbilical cord plasma highlighted potential differences in materno-foetal nutrient transfer and, probably, short-term changes associated with birth-related oxidative stress. Thus, it can be proposed that cord blood ^1^H NMR spectroscopy has considerable potential as a rapid and non-invasive tool to investigate the biochemical status in preterm delivery, including in VLBW infants. The identification of a number of VLBW-related biomarkers may also aid in evaluating the pre-parturition nutritional status of the neonate and in providing guidance to the design new strategies of supplementation with specific nutrients in VLBW newborns.
